# Australian vertebrate hosts of Japanese encephalitis virus: a review of the evidence

**DOI:** 10.1093/trstmh/trae079

**Published:** 2024-10-25

**Authors:** Kevin T Moore, Madelyn J Mangan, Belinda Linnegar, Tejas S Athni, Hamish I McCallum, Brendan J Trewin, Eloise Skinner

**Affiliations:** Centre for Planetary Health and Food Security, Griffith University, Gold Coast, QLD 4222, Australia; Centre for Planetary Health and Food Security, Griffith University, Gold Coast, QLD 4222, Australia; Centre for Planetary Health and Food Security, Griffith University, Gold Coast, QLD 4222, Australia; Harvard Medical School, Boston, MA 02115, USA; Department of Biology, Stanford University, Stanford, CA 94305, USA; Centre for Planetary Health and Food Security, Griffith University, Gold Coast, QLD 4222, Australia; CSIRO, Health and Biosecurity, Brisbane, QLD 4102, Australia; Centre for Planetary Health and Food Security, Griffith University, Gold Coast, QLD 4222, Australia; Department of Biology, Stanford University, Stanford, CA 94305, USA

**Keywords:** Australia, infectious disease reservoirs, Japanese encephalitis, vector borne diseases, viremia

## Abstract

Japanese encephalitis virus (JEV) transmission in temperate Australia has underscored a critical need to characterise transmission pathways and identify probable hosts of the virus. This systematic review consolidates existing research on the vertebrate hosts of JEV that are known to exist in Australia. Specifically, we aim to identify probable species involved in JEV transmission, their potential role as hosts and identify critical knowledge gaps. Data were extracted from studies involving experimental infection, seroprevalence and virus isolation and were available for 22 vertebrate species known to reside in Australia. A host competence score was calculated to assess the ability of each species to generate and sustain a viraemia. Based on the host competence score and ecology of each species, we find that ardeid birds, feral pigs and flying foxes have potential as maintenance hosts for JEV in the Australian context. We also note that domestic pigs are frequently infected during outbreaks, but their role as amplification hosts in Australia is unclear. Evidence to confirm these roles is sparse, emphasising the need for further targeted research. This review provides a foundation for future investigations into JEV transmission in Australia, advocating for enhanced surveillance and standardised research methodologies to better understand and mitigate the virus's impact.

## Introduction

Japanese encephalitis virus (JEV) is a mosquito-borne flavivirus responsible for the most common viral encephalitis in Asia, Japanese encephalitis.^1^ Although most human infections are asymptomatic, central nervous system infection by the virus can lead to symptoms such as fever, headache, vomiting, confusion, seizures, paralysis and, in severe cases, death.^2^ Approximately 3 billion people reside in at-risk areas throughout Southeast Asia and the Western Pacific, leading to an estimated 25 000 deaths in 2015.^1^ Beyond its impact on human health, JEV can also have significant economic implications, including reproductive losses in pigs and clinical encephalitis in horses during outbreaks.^3–5^ Mitigating the transmission of JEV requires a One Health strategy, integrating human, animal and environmental health efforts to address its complex transmission dynamics and reduce its societal burden.^4^

**Figure 1.**
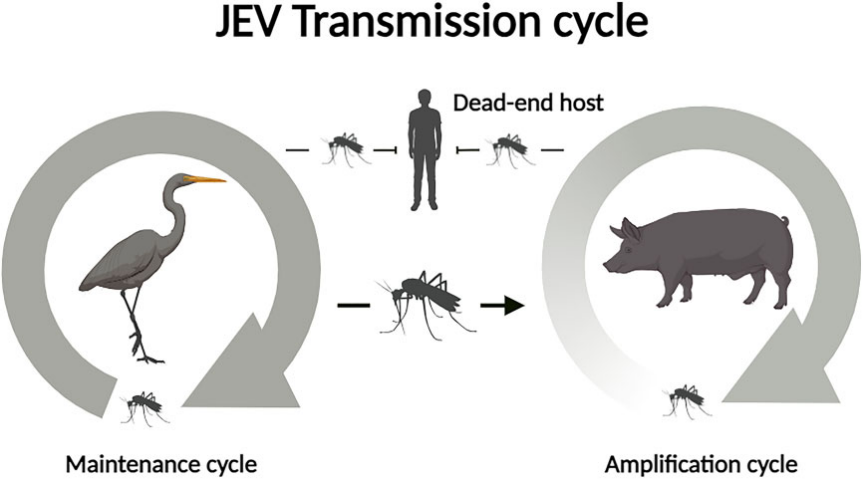
Transmission of JEV is dynamic with at least two different cycles. The maintenance cycle involves hosts that facilitate continued JEV transmission over time and between locations. Hosts in the amplification cycle become infected incidentally and sustain transmission for a limited time. Dead-end hosts (such as humans) do not contribute to onward JEV transmission. JEV, Japanese encephalitis virus.

JEV is a multi-host, multi-vector pathogen maintained in circulation between non-human vertebrates and *Culex* mosquitoes, with subsequent transmission to humans (Figure 1).^6^ More than 30 species of mosquitoes have yielded isolates of JEV,^7^ with the primary vector throughout most of Asia considered to be *Culex tritaeniorhynchus*, a mosquito that breeds in wetland rice fields near human settlements and domestic pig populations.^7^ Natural infection with JEV has been reported in a wide range of vertebrates including birds, bats, rodents, horses, cattle, snakes and pigs.^8^ Viraemia has been demonstrated through experimental infection in pigs and >12 bird species.^8^ Dead-end hosts (i.e. do not generate a viraemia) include humans, cows and dogs.^8^

Egrets and herons (Ardeidae family) are considered the primary hosts of JEV in Asia.^9-11^ Experimental infection and transmission studies have demonstrated the ability of Ardeidae species to generate viraemia sufficiently high to transmit JEV to competent vectors.^12-15^ The role of these species as hosts is based largely on extensive studies performed in Japan during the 1950s,^11,16-18^ however, few studies have confirmed their role, and the JEV transmission ecology in Japan is not necessarily applicable to other areas.^19^ In experimental studies, chickens, ducks and pigeons have shown viraemic titres in the range of those recorded for pigs, and may be an important alternative amplifying hosts due to their close proximity to humans.^7^ The primary amplifying host of JEV is the domestic pig.^20,21^ Pigs are frequently fed on by mosquitoes, develop high viral loads and are found in sufficient abundance to maintain transmission.^22^ Pigs are considered necessary for pre-epidemic amplification of JEV, although some human epidemics do occur in the absence of large pig populations.^21^

**Figure 2.**
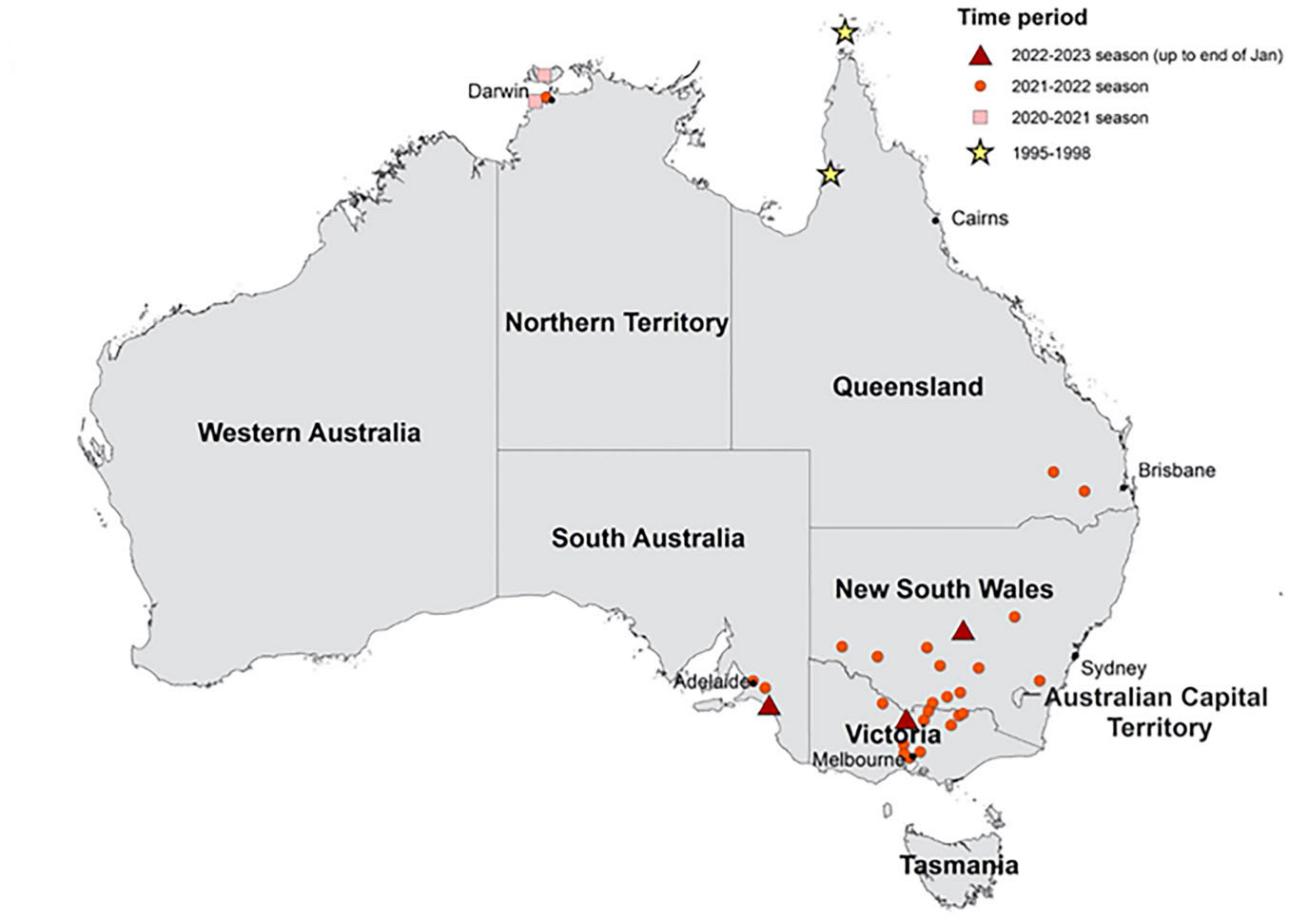
Residential location for 40 of 46 human JEV cases from the recent (2021–2023) outbreak and five of five cases of historical JEV outbreaks (1995 and 1998) in Australia. The location of symbols corresponds to the LGA residence of cases and may not reflect place of exposure. More than one case was reported for some LGAs. (Source: McGuinness et al. 2023.^27^). JEV, Japanese encephalitis virus. LGA, local government area.

In Australia, the hosts of JEV are largely unknown.^23^ Until recently, JEV in Australia was primarily considered a risk for people travelling to endemic areas in Asia.^23^ JEV first emerged in Australia in 1995 when an outbreak occurred on an island in the Torres Strait (Figure 2).^24^ Response to this outbreak was widespread and included the vaccination of 3340 people, vector control and viral surveillance within domestic vertebrates.^24,25^ Natural transmission of JEV was not reported in Australia again for >25 y.^26^ In the summer of 2021–2022, a large and widespread outbreak of JEV occurred across all but two states and territories in Australia (Figure 2).^26^

The 2021–2022 outbreak of JEV caused the deaths of seven people, infected >80 piggeries and resulted in a loss of >$A3 000 000 within the pig industry.^5,26^ The widespread nature of this outbreak raised questions about the introduction of JEV to Australia, the local hosts responsible for dispersal and whether the virus would become established and lead to future outbreaks.^26^

The aim of this review is to identify the existing evidence for vertebrate hosts of JEV within Australia, collate data across studies and identify gaps in our understanding of JEV transmission in Australia. Specifically, we synthesise data from studies on experimental infection, seroprevalence and virus isolation and calculate a host competence score for included species.

## Materials and Methods

We conducted a systematic review of the literature and extracted data from primary research studies. A detailed search strategy and inclusion criteria can be found in Supplementary Methods, Textbox S1 and Table S1. In brief, we included primary research studies of three data types: experimental infection, seroprevalence and virus isolation studies. We included experimental infection studies if they evaluated infection in native Australian species, migratory bird species that reside in Australia and imported or domestic species known to live in Australia (such as pigs, horses and water buffalo). We included seroprevalence and virus isolation studies if they took place in Australia.

To compare results of experimental infection studies across species and observations, we calculated a metric of host competence building on an approach used in other studies.^28,29^ We consider host competence to be a measure that quantifies a host's ability to replicate virus that allows for onward transmission to mosquito vectors. Higher host competence, representative of higher and longer lasting viraemia, would increase the probability of a mosquito vector acquiring JEV.^30,31^

We calculated the host competence scores in the following way. First, we calculated the mean duration of viraemia and mean peak viraemia across all individuals for each species from raw data extracted from the included experimental infection studies. Using these values, we generated a viraemia curve for each species by fitting a quadratic function and assuming that mean peak viraemia occurs halfway through the duration of viraemia; although not necessarily the case for most infections, we felt that choosing the halfway point was most appropriate for this analysis (Supplementary Methods). From these viraemia curves, we calculated the area under the curve (AUC) to allow comparisons between species with high, but short-lived viraemias with those with low, long-lasting viraemias. Finally, we multiplied the AUC by the proportion of individuals of that species that developed a viraemia to account for variation in susceptibility within species. This calculation thus includes the mean peak and mean duration of viraemia within a species and considers the proportion of individuals that had a viraemic response.

Australian vector competence was not considered within the host competence score because vector competence for JEV varies within Australian mosquito species as well as between virus genotypes tested in mosquito species of the same origin.^28^

## Results

### Systematic review

We identified 20 studies that included 22 vertebrate species present in Australia. Three studies were serosurveys and 17 were experimental infection studies. Most experimental infection studies took place outside of Australia (14/17; 82%). The three experimental infection studies conducted in Australia infected agile wallabies (*Macropus agilis*), black flying foxes (*Pteropus alecto*), brushtailed possums (*Trichosurus vulpecula*), Eastern grey kangaroos (*Macropus giganteus*), tammar wallabies (*Macropus eugenii*), Nankeen-night herons (*Nycticorax caledonicus*) and intermediate egrets (*Ardea intermedia*). Serosurveys tested for prior JEV infection in flying foxes, pigs and humans in Western Australia, the Northern Territory and the Torres Strait Islands. Humans and pigs were tested for JEV antibodies as part of outbreak investigations during the 1995 and 1998 JEV outbreaks in the Torres Strait Islands. We did not identify any studies that isolated virus from free-living vertebrates in mainland Australia, but one study isolated JEV from a sentinel pig in the Torres Strait in 1998.^32^ A summary of all the included studies is provided in Table S2.

### Seroprevalence studies

A total of 419 individuals from 11 species in Western Australia,^33^ the Northern Territory,^33^ the Torres Strait Islands and northern Queensland have been tested for antibodies to JEV (Table 1).^24,32^ Across the islands of the Torres Strait, seroprevalence was highest in pigs (*Sus scrofa*; 70%, 63/90), followed by horses (*Equus caballus*; 70%, 7/10) and dogs (*Canis lupus familiaris*; 63%, 10/16). Chickens (*Gallus domesticus*) did not generate a viraemia (0%, 0/6). In surveys conducted on the Cape York Peninsula in 1998, JEV antibodies were found in 65% (13/20) of domestic pigs. None of the 113 feral pigs tested positive for JEV Ab, although 90 of the 113 feral pigs exhibited cross-reacting flavivirus antibodies by plaque reduction neutralisation assay to Kunjin virus and Murray Valley Encephalitis virus.^32^ Surveillance of mega- and microchiroptera in Western Australia and the Northern Territory in 1998–1999 yielded potentially positive JEV antibodies detections in black flying foxes.^33^ However, again, cross-reactivity with Kunjin virus and Murray Valley encephalitis virus limited the meaningful interpretation of data from other bat species in this survey.^33^

**Table 1:** Summary of JEV seroprevalence studies conducted in Australia

Species	Location	Total tested	Total positive	Cross-reactive	Test	Reference
Domestic pig	Torres Strait inner islands	22	0	0	HI/PRNT	^24^
	Torres Strait outer islands	90	63 (70%)	0	HI/PRNT	^24^
	Cape York	40	13 (33%)	0	HI/PRNT	^32^
Feral pig	Cape York	113	0	90 (80%)	HI/PRNT	^32^
Horse	Torres Strait outer islands	10	7 (70%)	0	HI/PRNT	^24^
Dog	Torres Strait outer islands	16	10 (63%)	0	HI/PRNT	^24^
Chicken	Torres Strait outer islands	6	0	0	HI/PRNT	^24^
Black flying fox	Western Australia	5	1 (20%)	1 (20%)	ELISA/PRNT	^33^
	Northern Territory	2	1 (50%)	0	ELISA/PRNT	^33^
Little red flying fox	Western Australia	2	0	0	ELISA/PRNT	^33^
Yellow-bellied sheath-tailed bat	Western Australia	2	0	1 (50%)	ELISA/PRNT	^33^

Abbreviations: HI, haemagglutination inhibition assay; JEV, Japanese encephalitis virus; PRNT, plaque reduction neutralisation test.

### Viraemia from experimental infection

The 17 experimental infection studies included 22 Australian vertebrate species. All individuals were inoculated with strains of JEV from either genotype 1 (GI), genotype 2 (GII) or genotype 3 (GIII). The methods of inoculation and metrics used to measure viraemia differed between studies (Table 2). Studies prior to 1988 used LD_50_^10,13,34–36^ in mice to measure viraemia, whereas more recent studies used tissue culture infectious dose 50 (TCID_50_)^37–40^ and plaque-forming units (PFU).^41–47^ Across all studies, the median sample size for each species was five, with ducks and chickens having the largest sample sizes of 48 and 41, respectively.^45^

**Table 2:** Summary of JEV experimental infection studies with Australian vertebrate species, and mean host competence calculation from experimental infection data

Species	Maximum infectious dose (units) and method of inoculation	Mean peak viraemia titre (units)	Overall peak viraemia titre (units)	Mean duration (d)	Percentage viraemic (total tested)	Mean host competence^1^ (AUC of quadratic titre curve multiplied by percentage viraemic)	Location and study year
Agile wallaby (*Macropus agilis*)	5 (TCID_50_) subcutaneous	1.6 (TCID_50_)	1.6 (TCID_50_)	1	40% (5)	0.2	Australia, 2000^37^
Black flying fox (*Pteropus alecto*)	6.1 (TCID_50_) intravenous and mosquito	0 (PFU)	0 (PFU)	0	0 (10)	0	Australia, 2009^43^
Brushtailed possum (*Trichosurus vulpecula*)	5 (TCID_50_) subcutaneous	2.5 (TCID_50_)	3.0 (TCID_50_)	2.5	100% (4)	6.0	Australia, 2000^37^
Cattle egret (*Bubulcus ibis*)	5.8 (PFU) subcutaneous	2.9 (PFU)	3.7 (PFU)	4.5	100% (10)	10.4	USA, 2012^44^
Chicken (*Gallus domesticus*)	5.8 (PFU) subcutaneous; 1.3 (PFU) subcutaneous	3.4 (PFU)	5.0 (PFU)	3.0	65% (45)	6.5	USA, 2012^44^; USA, 2014^45^
Cow (*Bos taurus*)	9.2 (LD_50_) subcutaneous and mosquito	0 (PFU, LD_50_)	0 (PFU, LD_50_)	0	0 (13)	0	Thailand, 1974^41^; India, 1988^42^
Dog (*Canis lupus familiaris*)	Mosquito	0.2 (PFU)	0.5 (PFU)	1.0	33% (3)	0.1	Thailand, 1974^41^
Domestic duck (*Anas platyrhynchos domesticus*)	7.3 (PFU) subcutaneous	3.6 (PFU)	6.5 (PFU)	2.2	84% (86)	6.5	USA, 2012^44^; USA, 2014^45^; USA, 2019^46^
Eastern grey kangaroo (*Macropus giganteus*)	5 (TCID_50_) subcutaneous	0 (TCID_50_)	0 (TCID_50_)	0	0 (5)	0	Australia, 2000^37^
European starling (*Sturnus vulgaris*)	5.8 (PFU) subcutaneous	2.8 (PFU)	3.6 (PFU)	3.9	79% (9)	7.1	USA, 2012^44^
Great egret (*Ardea alba*)	5.8 (PFU) subcutaneous	3.8 (PFU)	4.2 (PFU)	4.0	100% (2)	12.7	USA, 2012^44^
		1.8 (LD_50_)	1.8 (LD_50_)	2.0	100% (1)	3.6	Japan, 1958^10^
Horse (*Equus caballus*)	Mosquito	1.6 (LD_50_)	2.7 (LD_50_)	2.0	67 (3)	2.2	USA, 1964^34^
House sparrow (*Passer domesticus*)	5.8 (PFU) subcutaneous	2.4 (PFU)	3.7 (PFU)	6.8	73 (18)	9.7	USA, 2012^44^
Intermediate egret (*Ardea intermedia*)	4.3 (LD_50_) subcutaneous	2.8 (LD_50_)	2.8 (LD_50_)	2.0	100 (1)	5.6	Australia, 1983^13^
Pig (*Sus scrofa domesticus*)	Mosquito (PFU); 6.9 (LD_50_) subcutaneous; 7.0 (TCID_50_); Mosquito	1.0 (PFU)	2.0 (PFU)	2.0	50% (2)	0.7	Thailand, 1974^41^
		2.3 (LD_50_)	3.5 (LD_50_)	2.3	100% (9)	5.2	Japan, 1959^11^
		3.4 (TCID_50_)	5.1 (TCID_50_)	3.3	100% (39)	9.7	Switzerland, 2016^38^; China, 2018^40^; USA, 2018^39^; India, 1969^35^; Australia, 2001^48^
Plumed egret (*Ardea plumifera*)	4.9 (LD_50_) mosquito (viraemia in mosquito tested)	1.7 (LD_50_)	1.8 (LD_50_)	2	100% (2)	3.4	Japan, 1958^10^
Ring necked pheasant (*Phasianus colchicus*)	5.8 (PFU) subcutaneous	0 (PFU)	0 (PFU)	0	0 (10)	0	USA, 2012^44^
Rock pigeon (*Columba livia*)	5.8 (PFU) subcutaneous	3.4 (PFU)	4.3 (PFU)	3.5	90% (10)	9.2	USA, 2012^44^
Nankeen night heron (*Nycticorax caledonicus*)	4.3 (LD_50_) subcutaneous	2.8 (LD_50_)	4.8 (LD_50_)	2.8	71 (7)	5.0	Australia, 1983^13^
Tammar Wallaby (*Macropus eugenii*)	5.0 (TCID_50_) subcutaneous	0 (TCID_50_)	0 (TCID_50_)	0	0 (5)	0	Australia, 2000^37^
Water buffalo (*Bubalus bubalis*)	Mosquito	0 (PFU, LD_50_)	0 (PFU, LD_50_)	0	0 (4)	0	India, 1968^41^; Thailand, 1974^41^

Abbreviations: AUC, area under the curve; JEV, Japanese encephalitis virus.

A 2-d-old duckling had the highest reported PFU viraemia at 6.4 PFU with a duration of 4 d,^45^ whereas a 7-wk-old pig had the highest TCID_50_ measured viraemia at 5.1 TCID_50_ with a duration of 3 d.^38^ Nankeen night herons had the highest LD_50_-measured viraemia at 4.8 LD_50_ with a duration of 3 d. The species with the longest mean duration was the house sparrow (6.8 d). Cows (*Bos taurus*), water buffalo (*Bubalus bubalis*), tammar wallabies, Eastern grey kangaroos, ring-necked pheasants (*Phasianus colchicus*) and black flying foxes did not produce a detectable viraemia; however, black flying foxes were able to infect feeding mosquitoes with JEV.^43^

### Host competence scores

Mean host competence is the term we use to succinctly refer to the host competence calculation, that is, the AUC of the quadratic viraemia curve multiplied by the proportion viraemic. Mean host competence describes the average host competence across all individuals of a species. Please refer to the supplemental methods for a full description of this calculation.

**Figure 3.**
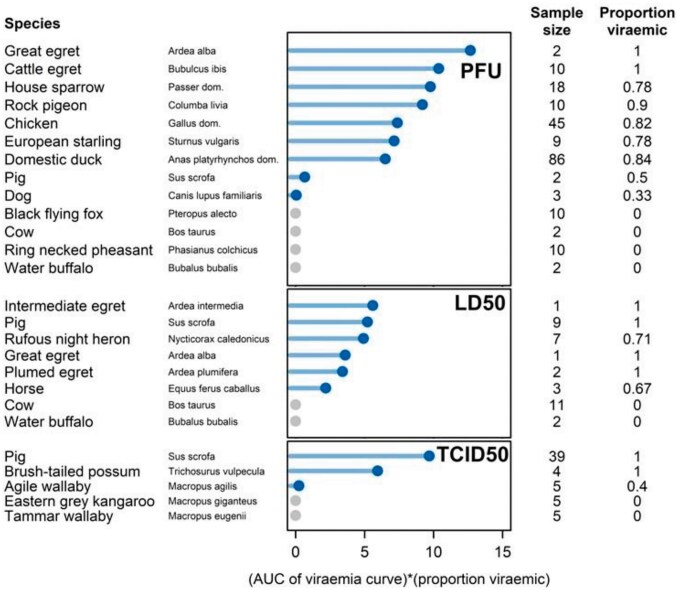
Mean host competence score, sample size and proportion viraemic for each species included in the review. Species are grouped by unit measure of viraemia (PFU, LD_50_, TCID_50_) and ranked from highest to lowest mean host competence score (solid circles). AUC, area under the curve; LD_50_, lethal dose 50; PFU, plaque-forming units; TCID_50_, tissue culture infectious dose 50.

All five Ardeidae species had high mean host competence scores, ranging from 3.4 to 5.6 for those measured with LD_50_ and from 10.4 to 12.7 for those measured with PFU (Figure 3). The great egret and cattle egret had the two highest mean host competence values across all species measured with PFU (10.4 and 12.7) and the intermediate egret had the highest mean host competence of those measured with LD_50_ (5.6) (Figure 3). The 27 pigs measured with TCID_50_ and LD_50_ had a high mean host competence (6.9 and 5.2, respectively), while the two pigs measured with PFU returned a low mean host competence of 0.7 (Figure 3).

Three other bird species, the rock pigeon (*Columba livia*; 9.2), European starling (*Sturnus vulagaris*; 7.1) and house sparrow (*Passer domesticus*; 9.7), have mean host competence scores similar to the two Ardeidae species included in the same study (great egret [*Ardea alba*; 12.7] and cattle egret [*Bubulcus ibis*; 10.4]) (Figure 3).^44^ The agile wallaby (*Macropus agilis*; 0.2), dog (*Canus lupus familiaris*; 0.1) and horse have low mean host competence scores attributed to low titres and short durations of viraemia (Figure 3).

## Discussion

JEV is sustained in the environment by the interactions between multiple vector and vertebrate host species.^49^ Not all vertebrate host species contribute equally to transmission, with some species considered more broadly as maintenance hosts, amplification hosts or dead-end hosts.^50,51^ Vertebrate host species integral to the persistence of JEV—whereby their absence would lead to a reduction or elimination of JEV within the community—are considered maintenance hosts. By contrast, vertebrate species that contribute to JEV transmission, but only for short periods of time, are considered amplification hosts.^52^ Those species that cannot infect susceptible vectors are considered dead-end hosts. In the Australian context, the role of different vertebrate species as maintenance, amplification or dead-end hosts is unknown, but has important implications for the management and mitigation of future transmission. Here, we synthesise our findings of Australian species in the context of these different host definitions, but acknowledge that understanding JEV transmission in Australia is incomplete and with new data these potential species roles could change.

### Possible maintenance hosts of JEV in Australia

In endemic areas of JEV transmission (such as Japan), the Ardeidae family of birds have long been considered the key maintenance hosts of JEV because they generate long-lasting viraemias, are frequently and preferentially fed upon by vectors, have regular population turnover and consistently demonstrate JEV exposure in free-living populations.^9-11^ In Australia, there are 12 species of the Ardeidae family, for which experimental infection data were available for five species.^10,13,44^ These five species had consistently high mean host competence scores (3.4–12.7 mean host competence) compared with other non-Ardeidae species experimentally infected with JEV.^10,13,44^ Additionally, there is strong ecological support for Ardeidae species as maintenance hosts based on their high abundance and colonial breeding in areas of JEV transmission in Australia and their wide distribution across the country.^53^

Although the host competence and ecology of Ardeidae species provides strong evidence as potential maintenance hosts of JEV in the Australian landscape, there is limited evidence demonstrating the interactions between vectors, Ardeidae species and JEV in Australia. In a meta-analysis of all bloodmeal studies in Australia, only one *Culex annulirostris* (the primary JEV vector in Australia) was reported to have fed on Ardeidae.^28,54^ Yet these species are considered the primary hosts of Murray Valley encephalitis virus and Kunjin virus, two flaviviruses related to JEV circulating in Australia, which suggests Australian vectors are frequently interacting with Ardeidae species.^55,56^ More studies are needed to identify previous JEV exposure in Ardeidae populations, mosquito-feeding patterns across space and time and the immunological implications of previous Murray Valley encephalitis virus infection. Without these studies, our understanding of Ardeidae species in past or future JEV outbreaks is incomplete.

Non-Ardeidae bird species have been posited as maintenance hosts of JEV in a number of other studies.^9,46,50^ The original investigations of JEV hosts in Japan noted that although Ardeidae species were their primary focus, this was not intended to exclude other species from consideration in JEV transmission.^11^ In fact, experimental infection studies in the last decade identified high viraemias in rock pigeons, house sparrows and European starlings.^44^ When comparing host competence scores calculated in this review, these three species have scores below the Ardeidae species but higher than pigs. There is evidence of natural exposure to JEV in these three species in countries where JEV transmission is endemic. Specifically, in wild pigeons in India, JEV antibodies were prevalent throughout the year.^57^ In Thailand, a small percentage of wild tree sparrows had antibodies to JEV,^41^ and in Japan, 20–37% of wild-caught sparrows had antibodies to JEV.^58^ The ecology of these species also supports their potential as maintenance hosts; they congregate in high numbers, are short-lived and have moderate clutch sizes allowing for the availability of a susceptible population.^59-61^ In Australia, these species have not yet been investigated as potential maintenance hosts, and there are an absence of data on mosquito-feeding patterns for these species, as well as previous exposure to JEV.

Flying foxes (genus *Pteropus*) may serve as maintenance hosts for JEV due to three key factors: (i) they can transmit JEV to mosquitoes^43^; (ii) evidence suggests prior exposure to JEV in Australia^33^; and (iii) Australian JEV vectors are known to feed on them, confirming a potential transmission pathway.^28^ Recent studies in Indonesia, where the same JEV genotype (GIV) responsible for the Australian outbreak circulates, revealed that 5.6% of bats sampled (21/373) tested positive for JEV using RT-PCR,^62^ and 4.2% of *Pteropus* bats (3/70) showed previous exposure to the virus.^63^ These studies indicate that JEV can be transmitted from mosquitoes to bats but does not confirm that bats are transmitting the virus further. The evidence in Australia is mixed. The only seroprevalence study estimated that <3% (3/119) of bats had prior exposure to JEV, but the authors note a high potential for cross-reactivity to other closely related flaviviruses.^33^ An Australian experimental infection study found no detectable viraemia in any of the 10 black flying foxes included, but at least three of these individuals were able to infect susceptible *Cx. annulirostris* vectors.^43^ Bats are known to have unique immune systems, which has been observed in the experimental infection of JEV in Microchiropteran species^64^; tricoloured bats (*Perimyotis subflavus*; native to North America) were capable of maintaining viraemia after induced hibernation periods.^64^ Australia is home to >90 species of bat that inhabit diverse ecological niches, travel large distances and gather in dense communities.^65^ Although there is potential that some of these species could maintain JEV transmission, more evidence is needed to identify their capacity to do so.

In Australia there are two distinct swine populations that could have differing transmission roles for JEV: feral pigs and domestic pigs. Feral pigs in Australia are descendants of domestic pigs that have returned to a wild state. These pigs roam freely and have adapted to a variety of natural habitats across the country. Australia has a feral pig population of >24 million distributed across at least 45% of the continent.^66^ This large and widely distributed population is conducive to participation in the maintenance cycle, as opposed to domestic pigs, which live in isolated populations with minimal to no movement. Also, some of these feral pig populations co-occur with JEV vectors that are active year-round in northern Australia.^28^ Feral pigs accounted for 82% of vector bloodmeals identified (in the absence of domestic pigs) at the location that yielded the first Australian mainland isolate of JEV from mosquitoes.^67^ Wild boar sera were collected in Japan and 86% were positive for neutralising Ab against JEV, even in winter.^68^ The prevalence of JEV antibodies in wild boar has been cited as 83% in Japan,^69^ 66% in South Korea,^70^ 32% in Indonesia^71^ and 15% in Singapore.^72^ In March 2022, as part of a routine animal health survey, a small number of feral pigs in northern Australia tested positive for JEV,^73^ and retrospective serology from 2020–2021 show evidence of exposure in three feral pigs in northern Australia.^73^

Data from mosquito blood meal analyses have also found support for vector–pig interactions. There are eight JEV vector species in Australia that were found to feed on feral pigs.^74^ The hypothesised primary vector of JEV in Australia, *Cx. annulirostris*, was found to feed on feral pigs in Badu, northern Queensland, a location with previous JEV transmission.^67^ In general, feral pigs are not the primary bloodmeal source for JEV vectors in Australia, even in areas with dominant feral and domestic pig populations.^28^ However, JEV has been reported to spread in areas with low porcine feeding rates, as demonstrated by Hall-Mendelin et al. in northern Australia, which could suggest the importance of other hosts in the transmission cycle.^67^ Theoretically, feral pigs in northern Australia could participate in a maintenance transmission cycle, but this prospect has yet to be adequately investigated.

### Possible amplification hosts for JEV in Australia

In JEV-endemic regions of Asia, domestic pigs act as amplifying hosts and play a central role in transmission in certain areas.^22,50^ An amplifying host increases the level of circulating virus, which leads to pathogen pressure on humans.^49^ In many locations across Asia, domestic pigs fit this definition because they live in dense populations near humans and develop high viraemias that infect many mosquitoes.^22^ Likewise, after the 1995 outbreak in the Torres Strait, researchers noted the proximity of domestic pigs and *Cx. annulirostris* breeding sites to human residences.^24^ However, during the 1998 outbreak in the Torres Strait and Northern Peninsula Area (NPA) of Cape York, the relationship between domestic pigs and human cases was less clear.^32^ A successful vaccination campaign in the Torres Strait after the 1995 outbreak, along with unique climate factors and a lower percentage of NPA households keeping pigs (10% in NPA compared with 50% on Badu Island in the Torres Strait), probably contributed to reduced transmission to humans in these areas.^25,32^

Most domestic pigs in Australia are raised in controlled environments, primarily in large commercial piggeries. These pigs are managed for agricultural production and are not free-ranging. Although domestic pigs have demonstrated strong host competence (5.2 [LD_50_], 6.9 [TCID_50_] and 0.7 [PFU]) and are frequently infected during outbreaks,^32^ their populations in Australia are isolated and we do not have evidence that Australian domestic pigs act as amplification hosts in an ongoing JEV transmission cycle. Therefore, primarily due to a lack of evidence about the role of Australian domestic pigs in the transmission of JEV, we consider domestic pigs to be a spillover host. We consider a spillover host to be a species that can transmit the virus, but whose biological/ecological characteristics limit their ability to independently maintain transmission (i.e. in the absence of other host species). We have evidence of repeated exposure in domestic pigs, but no evidence of onward transmission and little evidence of pathogen pressure on humans. During the widespread JEV outbreak in Australia in 2021–2022, some commercial piggeries reported JEV infections over multiple weeks, which led to devastating losses among their herds and threatened their closure.^5^ However, it is unclear whether this sustained transmission was due to a transmission cycle with domestic pigs as the primary host or whether an ongoing transmission cycle in the nearby natural habitat led to repeated introductions into the domestic pig herd.^5^ Additionally, the Australian commercial pork industry did not observe any direct transmission of JEV between domestic pigs during the 2021–2022 outbreak in Australia.^5,75^

After the 2021–2022 outbreak in southeast Australia, a serosurvey for JEV antibodies was conducted among the human population in northern Victoria.^76^ Contact with domestic pigs was not associated with JEV IgG-seropositivity, but most piggery workers (many of whom live onsite) were ineligible for this study because of previous JEV vaccination.^76^ Overall, domestic pigs in Australia are competent hosts and they are frequently infected when JEV is circulating. The link between the maintenance cycle of JEV and domestic pigs has not been identified, and the degree to which domestic pigs amplify JEV and increase the risk of JEV transmission to Australians requires further evaluation.

### Species with unknown roles

Not enough data are available to distinguish the potential role of domestic ducks and chickens as maintenance or amplification hosts. Although the host competence scores for ducks indicate their potential as hosts (6.5 mean host competence), there are two caveats that must be considered. First, the individuals identified in this review are domestic ducks (*Anas platyrhynchos domesticus*), and their results may not extrapolate to other wild species in the genus *Anas*. Second, the majority of the individuals in the original experimental infections were newly hatched (<3 wk old; 60/86, 70%) and, as Cleton et al. demonstrated, viraemia is a function of age in young ducks, where younger ducks (<20 d) generate a much higher viraemic response than older ducks.^45^ Within our dataset, the mean host competence for ducks aged >20 d is 1.9; the mean host competence for ducks aged <20 d is 9.2. This difference indicates that adult domestic ducks might be less competent hosts than young ducks (and less competent than other bird species), but it is not clear how this would affect JEV transmission.

The role of domestic ducks as maintenance hosts therefore remains unknown. On one hand, in JEV-endemic regions, both wild and domestic duck species are frequently infected. In seroprevalence studies, significant numbers of ducks were found to have antibodies to JEV in India (*Anas clypeata, Anas crecca, Aythya fuligula, Anas strepera*)^57,77^ and Thailand (*Anas platyrhynchos domesticus*).^41^ In Indonesia, antibodies against JEV were found in ducks (20.6%), with no difference in seroprevalence between domestic ducks kept closely with pigs compared with those reared without pigs.^71^ In Japan, 85.9% of wild ducks were positive for JEV antibodies (*Anas poecilorhyncha, Anas platyrhynchos, Ana acuta, Anas Penelope*).^78^ On the other hand, unlike pigs, the epidemiological significance of domestic ducks living in proximity to humans has not been studied.^79^ Death associated with natural JEV infection is not observed among domestic ducks, and therefore JEV outbreaks might be ignored.^47^

Like domestic ducks, chickens have been successfully used in JEV mosquito transmission studies, suggesting that chickens may exhibit sufficient viraemia and receptivity to infection to participate in the natural transmission cycle.^50^ However, in an experimental infection study conducted in 1951, chickens were deemed relatively unsusceptible, with the virus detected in only 4/12 (33%) at low titres^17^ (data not included in host competency calculations because of incompatible methods). Overall, chickens have a strong mean host competence of 6.5, but only 65% (34/52) of individuals developed a viraemia, and all of these viraemic chickens were young individuals, illustrating that viraemia is also a function of young age in chickens.^45^

### Dead-end hosts

Eight of the species included in this review developed no more than trace viraemia and, unlike flying foxes, did not demonstrate the ability to infect mosquitoes. In the experimental infection study with macropods, two agile wallabies and two tammar wallabies developed trace viraemias and one Eastern grey kangaroo generated a low-level antibody response.^37^ The lack of viraemia generated by these three species indicates that they are not likely to contribute to transmission; however, more replication is needed to confirm this. Furthermore, in mosquito-feeding studies across Australia, macropods make up a large proportion of bloodmeals in JEV vectors, including *Cx. annulirostris* and *Cx. sitiens*.^28^ This suggests that if infected vectors were to feed on susceptible macropods, the macropods could play a role as dilution hosts.^80^ However, exhaustive evaluation of this hypothesis would require studies on transmission pathways and exposure before it could be confirmed. It is plausible that other macropod species differ in their response to JEV, given that macropods play a role in other arboviral transmission in Australia.^81,82^

In endemic areas of JEV transmission, horses have often been considered dead-end hosts alongside humans.^83^ However, closer inspection of this information reveals limited and conflicting data. A single experimental infection study, with a small sample size (n=3), found that horses developed a low mean peak viraemia (1.6 LD_50_).^34^ In the same study, Gould et al. demonstrated experimental horse-to-chick and horse-to-horse transmission via mosquitoes.^34^ Horses do experience morbidity from JEV infection with fatality reported between 5 and 30%,^83^ and infections in unvaccinated horses were reported to be as high as 73%.^84^ Consequently, countries such as China, Japan, Korea and India practise JEV vaccination of their horses, with the result that equine infections are now rare and predominantly subclinical in these countries.^3,85^ However, horses, unlike pigs, do not appear to significantly contribute to JEV transmission to mosquitoes, and their smaller population size, slow turnover and extended lifespan likely limit their role as hosts.^86^

The prevailing theory that cattle are dead-end hosts is supported by experimental infection studies showing no detectable viraemia in inoculated cattle.^36,41^ Several studies report high seroprevalence (21–51%) among cattle in Asia,^87-89^ indicating that JEV is transmitted to cattle and that they generate an immune response. Boyer et al. found that in Cambodia, JEV vectors demonstrated a preference to cattle over chickens and humans, with pigs as the secondary choice.^90^ Overall, cattle do not demonstrate disease or generate a viraemia, and therefore are most likely dead-end hosts. Similar to macropods, a high proportion of bloodmeals in northern Australia came from cattle, suggesting that they too could play a role as dilution hosts, depending on the transmission and population dynamics of both vectors and cattle.^54^

Several other species of free-ranging vertebrates (water buffalo, dogs and ring-necked pheasants) were included in this review and are considered dead-end hosts for JEV transmission. Although these species may become infected with JEV and demonstrate a high prevalence of antibodies, they develop low viraemic responses.^6^ A small number of water buffalo (n=2) were experimentally infected and neither developed a viraemia.^35^ A single experimental infection study on dogs failed to detect more than a trace viraemia in one of the three (33%) of the infected individuals. In Cambodia, JEV seroprevalence was 35% in dogs ^91^ and a Malaysian study showed that 80% of dogs had JEV antibodies (commercial IgG ELISA).^88^ While rats and mice experimentally develop HI antibodies to JEV^92^ and 45.8% of rats sampled in south China were positive for JEV-reactive IgG antibodies,^93^ rodents are not known to play a role in transmission of JEV in Asia.^93^ Snakes have rarely demonstrated viraemia; however, some species show a high prevalence of JEV antibodies.^94-96^ Interestingly, ring-necked pheasants were the only bird species in this review that did not generate a viraemia.^44^ Nemeth et al. remark that two species of the order Galliformes (i.e. chickens and ring-necked pheasants) generate low to undetectable viraemia.^44^

### Limitations and future research

This review is limited by the scarcity of research on Australian hosts. Specifically, the limited number of species studied to date presents a major knowledge gap for the hosts of JEV in Australia, especially when considering the diversity of Australian species across habitats and climates. Additionally, we could not directly compare the host competence of species due to inconsistent methods, poor reporting and small sample sizes. Each study varied in the way they measured virus titres, even when using the same general approach.

Experimental infection studies aim to illuminate one step in the transmission cycle (host viraemia) and interpretation of their results should not include assumptions about subsequent steps. Most experimental infection studies in this review, for example, did not expose viraemic hosts to feeding mosquitoes to determine onward transmission. Furthermore, some mosquito vectors are specialist feeders, and although a vertebrate can develop a high viraemia, they may not be fed upon by competent JEV vector species.

With regard to the Australian seroprevalence surveys, certain species were more likely to be targeted than others. The surveys that took place after the 1995 and 1998 outbreaks in northern Australia focused on the communities that were affected and did not contend with the idea of more widespread transmission of JEV. They did not sample wild bird populations, marsupials or bats. The lone Australian seroprevalence survey of bats did not include randomly sampled bats and was limited by cross-reactivity with other flaviviruses, which underscores the potential that other seroprevalence studies may also experience cross-reactivity to Kunjin and Murray Valley encephalitis virus, both of which circulate endemically in Australia.

The experimental infection studies in this review used strains of JEV from the GI, GII or GIII genotypes. Most studies used only one strain of JEV, some used multiple strains of the same genotype, while only one study used two genotypes in the same study. Xiao et al. used the same methods to experimentally infect piglets with either GI or GIII.^40^ They found little difference between the genotypes, with a piglet inoculated with GI producing the highest viraemia. The pooled results from all experimentally infected piglets would indicate that GIII produces a higher viraemic response in piglets. The 2021–2022 Australian outbreak was caused by the GIV strain, but no experimental infection studies have been conducted with GIV. Therefore, we caution labelling one genotype more transmissible than another because the differences between species and genotypes could be due to experimental design or other biases.

Australian mosquito species likewise differ in their ability to transmit JEV, with each species varying in its host-feeding patterns, vector competence and population dynamics, among other factors.^28^ A review by van den Hurk et al. demonstrated that *Cx. annulirostris* is the primary vector species in Australia.^28^ Along with pigs and birds, *Cx. annulirostris* readily feed on marsupials, humans and other placental mammals, a generalist feeding strategy that could result in a variety of host species being exposed to JEV.^28^ Future studies should investigate vector–host interactions across space and time, particularly at the interface between piggeries and wild bird populations.^28^

## Conclusions

This review set out to synthesise the existing evidence on Australian vertebrate hosts of JEV. Ardeidae birds and other non-Ardeidae bird species, along with flying foxes, emerged as potential maintenance hosts, but the absence of comprehensive data on their interaction with vectors and JEV exposure in Australia hinders a conclusive determination. Domestic pigs in Australia are frequently infected (particularly during the 2021–2022 outbreak), but evidence of their role in onward transmission is lacking. The roles of domestic ducks, chickens and other groups such as macropods, horses and cattle are unclear, with some evidence pointing towards their designation as dead-end hosts. The complexity of host–virus–vector interactions, influenced by ecological, biological and environmental factors, underscores the challenge of identifying definitive host roles.

In conclusion, our systematic review highlights significant gaps in the current understanding of the host roles in the Australian JEV transmission cycle. Despite the identification of 22 Australian vertebrate species in experimental infection studies, the lack of a comprehensive ecological context limits definitive conclusions about their roles as maintenance or amplification/spillover hosts. Future research should focus on expanding the range of species studied, particularly marsupials and birds, with standardised methodologies to facilitate accurate comparisons. Enhanced JEV surveillance, including year-round monitoring, especially in northern Australia, is essential to understand the virus's maintenance and introduction patterns. Only through such comprehensive and integrated research efforts can we hope to fully elucidate the transmission dynamics of JEV and develop effective strategies for its control and management in Australia.

## Supplementary Material

trae079_Supplemental_File

## Data Availability

The code and data are available via Github at https://github.com/ThaboMoore/JEV-Host-Review.git.
